# Role of transforming growth factor-β1 pathway in angiogenesis induced by chronic stress in colorectal cancer

**DOI:** 10.1080/15384047.2024.2366451

**Published:** 2024-06-10

**Authors:** Jie Zhang, Yao-Tiao Deng, Jie Liu, Lu Gan, Yu Jiang

**Affiliations:** a Department of Oncology, The First Affiliated Hospital of Chongqing Medical University, Chongqing, People’s Republic of China; b Department of Medical Oncology, Cancer Center, West China Hospital, Sichuan University, Chengdu, People’s Republic of China

**Keywords:** Chronic stress, norepinephrine, transforming growth factor-β1, hypoxia inducible factor-1α, angiogenesis

## Abstract

**Background:**

Chronic stress can induce stress-related hormones; norepinephrine (NE) is considered to have the highest potential in cancer. NE can stimulate the expression of hypoxia-inducible factor-1α (HIF-1α), which is associated with vascular endothelial growth factor (VEGF) secretion and tumor angiogenesis. However, the underlying mechanisms are poorly understood.

**Methods:**

Tumor-bearing mice were subjected to chronic restraint stress and treated with normal saline, human monoclonal VEGF-A neutralizing antibody bevacizumab, or β-adrenergic receptor (β-AR) antagonist (propranolol). Tumor growth and vessel density were also evaluated. Human colorectal adenocarcinoma cells were treated with NE, propranolol, or the inhibitor of transforming growth factor-β (TGF-β) receptor Type I kinase (Ly2157299) *in* *vitro*. TGF-β1 in mouse serum and cell culture supernatants was quantified using ELISA. The expression of HIF-1α was measured using Real time-PCR and western blotting. Cell migration and invasion were tested.

**Results:**

Chronic restraint stress attenuated the efficacy of bevacizumab and promoted tumor growth and angiogenesis in a colorectal tumor model. Propranolol blocked this effect and inhibited TGF-β1 elevation caused by chronic restraint stress or NE. NE upregulated HIF-1α expression, which was reversed by propranolol or Ly2157299. Propranolol and Ly2157199 blocked NE-stimulated cancer cell migration and invasion.

**Conclusions:**

Our results demonstrate the effect of NE on tumor angiogenesis and the critical role of TGF-β1 signaling during this process. In addition, β-AR/TGF-β1 signaling/HIF-1α/VEGF is a potential signaling pathway. This study also indicates that psychosocial stress might be a risk factor which weakens the efficacy of anti-angiogenic therapy.

## 1. Introduction

Angiogenesis is one of the hallmarks of the occurrence and development of solid tumors, during which vascular endothelial growth factor A (VEGF-A) plays a critical role.^1^ Bevacizumab, a human monoclonal VEGF-A neutralizing antibody, was the first anti-angiogenesis drug approved by the Food and Drug Administration (FDA) for metastatic colon cancer. Although bevacizumab provides a clinical benefit to a subset of patients, others do not respond or gradually develop resistance.^2^ No clinical benefit has been shown in monotherapy with bevacizumab but has been demonstrated in combination with chemotherapy or immunotherapy.^3,4^ However, bevacizumab alone has shown promising antitumor activity in animal models.^5^ The possible mechanisms underlying the differences between patients and animals warrant further exploration.

The majority of cancer patients suffer from enduring negative emotions, including anxiety and depression, and are in a state of chronic stress.^6,7^ Numerous epidemiological studies have identified chronic stress as a pivotal element in oncogenesis and progression.^8,9^ Stress-related hormones (SRHs), including epinephrine, norepinephrine, and glucocorticoids etc., are released in response to chronic stress, which mediates the hypothalamic-pituitary-adrenal axis and sympathetic nervous system.^10,11^ Notably, norepinephrine (NE) is regarded as the most influential tumor-related SRH and is widely used to simulate chronic stress.^12^

Previous studies have reported that NE can act on various cancer cells, such as breast cancer, ovarian cancer, colon cancer, lung cancer, and prostatic cancer, upregulate VEGF, IL-6, IL-8, and matrix metalloproteinase, induce epithelial – mesenchymal transition (EMT), and further increase migration or invasion through β-adrenergic receptor (β-AR) activation. Propranolol, an antagonist of β-AR, can block the NE effect.^13–18^ Chronic stress is known to facilitate the growth, progression, and metastasis of human tumor xenografts. Propranolol was also found to counteract the impacts of chronic stress.^14–16,19^ In our previous studies, we demonstrated that NE induced cancer cells to secrete VEGF, which in turn promoted tumor angiogenesis and further attenuated the anti-angiogenic efficacy of sunitinib, a small-molecule targeted drug.^15,17^ Theoretically, norepinephrine is stimulated in chronic stress, and VEGF is secreted, impairing the efficacy of bevacizumab. Chronic stress seems to be a potential promoter of tumor angiogenesis and may influence the effect of bevacizumab. However, this phenomenon has not been proven in monoclonal antibodies and the underlying mechanisms remain to be elucidated.

Glucocorticoids and catecholamines can stimulate the secretion of TGF-β1,^20,21^ which is an independent prognostic risk factor for cancer patients.^22,23^ HIF-1α, a downstream signaling molecule of TGF-β1, can be activated upon the interaction of TGF-β1 with its receptor I (TGF-βRI) and further mediates VEGF secretion.^24,25^ Inhibition of TGF-βRI has been shown to be associated with lower VEGF levels and less angiogenesis, invasion, and metastasis in hepatocellular carcinoma.^26,27^ The attenuated activity of TGF-β1 contributes to reduced tumor hypoxia.^28^ Moreover, at the protein level, decreased expression of HIF-1α and downregulated VEGF levels occurred in parallel in a time-dependent manner.^29^ Therefore, TGF-β signaling and HIF-1α may be two significant factors involved in chronic stress-related tumor angiogenesis.

This study aimed to verify the efficacy of bevacizumab in the treatment of chronic stress. Another purpose of this study was to explore the role of TGF-β1 and HIF-1α and investigate its potential mechanism.

## 2. Results

### 2.1. Chronic restraint stress promoted tumor growth and weakened the efficacy of bevacizumab

**Figure 1.**
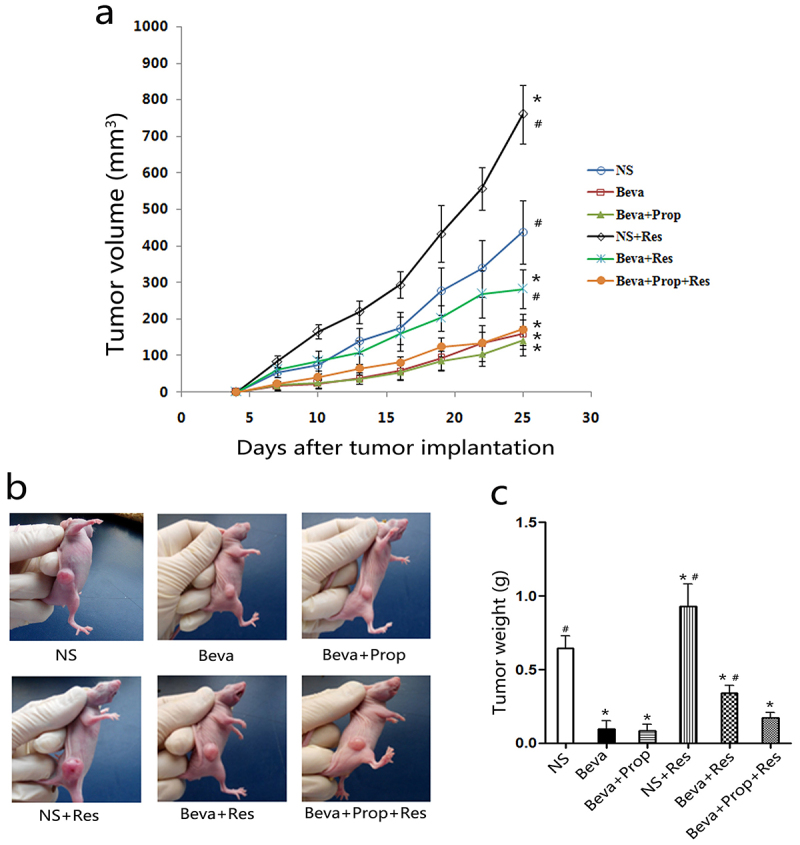
Chronic restraint stress promoted tumor growth and weakened the efficacy of bevacizumab.

To confirm chronic stress could impair the efficacy of bevacizumab *in vivo*, we established tumor model and CRS model in mice to observe the changes of tumor, according to tumor volume and tumor weight. When tumors in the right flank were palpable, the mice were administered normal saline, bevacizumab, propranolol, or CRS for 21 days. As shown in Figure 1, bevacizumab treatment (Beva) led to a flatter tumor volume curve (Figure 1a), smaller tumor volume (Figure 1b), and lighter tumor weight (Figure 1c), while chronic restraint (NS+Res) resulted in a larger volume and heavier weight compared to the control group (NS). Moreover, CRS accelerated the tumor growth rate in mice treated with Beva (Beva+Res) compared to the Beva group. This indicated that bevacizumab was effective in HT-29 cells *in vivo* and that the efficacy of bevacizumab was impaired by CRS. However, the tumor volume and tumor weight in the Beva+Prop+Res group were lower than those in the NS and Beva+Res groups. There were no significant differences between Beva, Beva+Prop, and Beva+Prop+Res. This means that bevacizumab was not affected by propranolol, and that propranolol could block the effect of CRS. This means that β-AR is involved in this process as well.

Tumor-bearing mice received normal saline, bevacizumab, propranolol, or CRS for 21 d. The tumor volume curve (a) and representative tumor-bearing mice in each group (b) indicated that CRS accelerated tumor growth and attenuated the efficacy of bevacizumab. Propranolol blocks the effects of CRS. Similar results were observed for tumor weight (c). *p < .05, vs. the NS group; #*p* < .05, vs. the Beva group.

### 2.2. Chronic stress stimulated angiogenesis in HT-29 model and was blocked by propranolol

**Figure 2.**
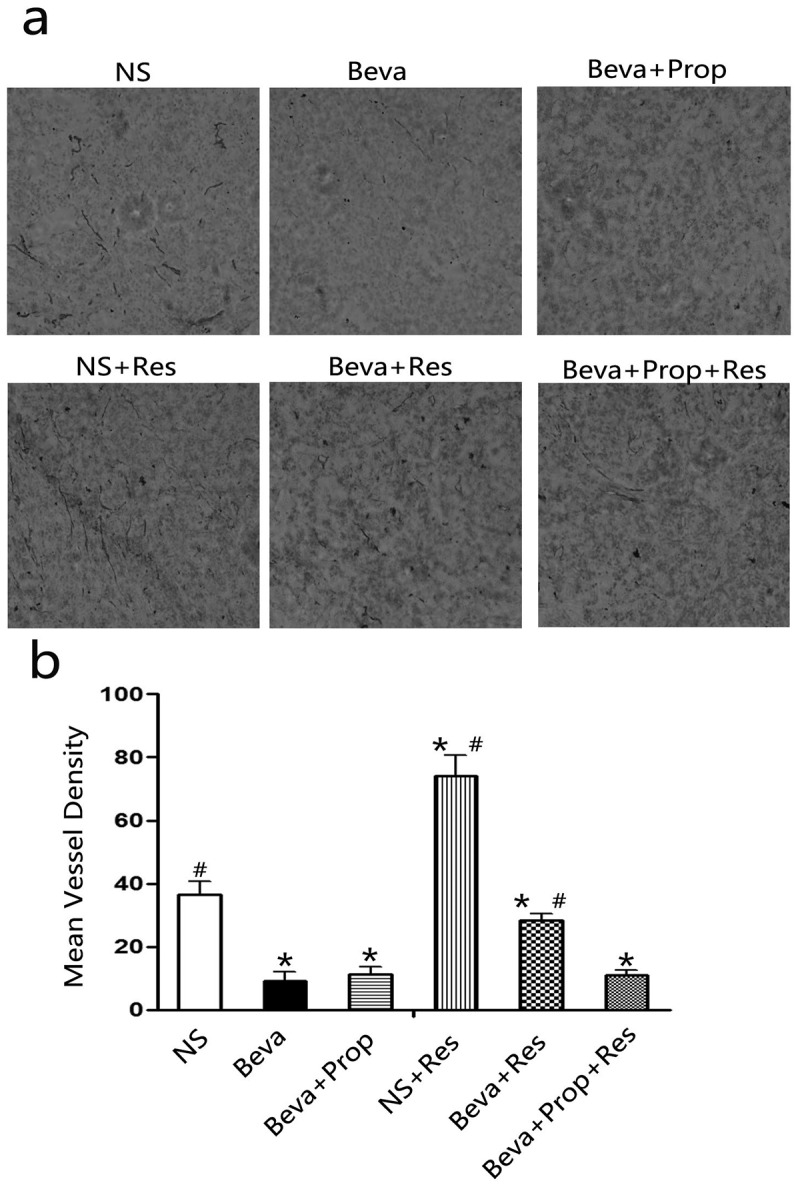
Propranolol inhibited chronic restraint stress-stimulated angiogenesis *in* *vivo*.

Bevacizumab is a humanized monoclonal antibody against VEGF-A, a key factor inducing the formation of blood vessels (angiogenesis) in tumors, and the number of vessels in tumor tissues could reflect the efficacy of bevacizumab to some degree. To test the hypothesis that CRS could stimulate angiogenesis *in vivo* and further impair the efficacy of bevacizumab, tumor tissues from the tumor-bearing mice were sliced into cryosections and stained with a murine endothelial cell marker (CD31) to quantify the mean vessel density (MVD) counts, which represent the number of blood vessels. Figure 2a shows the representative photographs of the cryosections in each group. As shown in Figure 2b, MVD counts significantly increased in the NS + Res group. Bevacizumab inhibited tumor angiogenesis because of the minimum MVD, but higher MVD counts were observed in the Beva+Res group. However, the MVD counts were lower in the Beva+Prop+Res group than in the Beve+Res group. No statistical significance was observed when comparing Beva, Beva+Prop, and Beva+Prop+Res. The results not only verified the efficacy of bevacizumab but also confirmed the role of CRS in angiogenesis. This also indicated that the effect of CRS is dependent on β-AR.

Frozen sections of HT-29 tumor tissues from mice that received normal saline, bevacizumab, propranolol, or CRS were stained with an anti-CD31 antibody (a) (brown; magnification, ×200). MVD was estimated by averaging the total number of immunoreactive endothelial cells from five random fields (b). Bevacizumab decreased MVD, and CRS significantly increased MVD. The efficacy of bevacizumab was attenuated by CRS, whereas the efficacy of CRS was reversed by propranolol. *p < .05, vs. the NS group; #*p* < .05, vs. the Beva group.

### 2.3. The effects of chronic restraint stress and norepinephrine on TGF-β1 secretion and TGF-βRI expression

**Figure 3.**
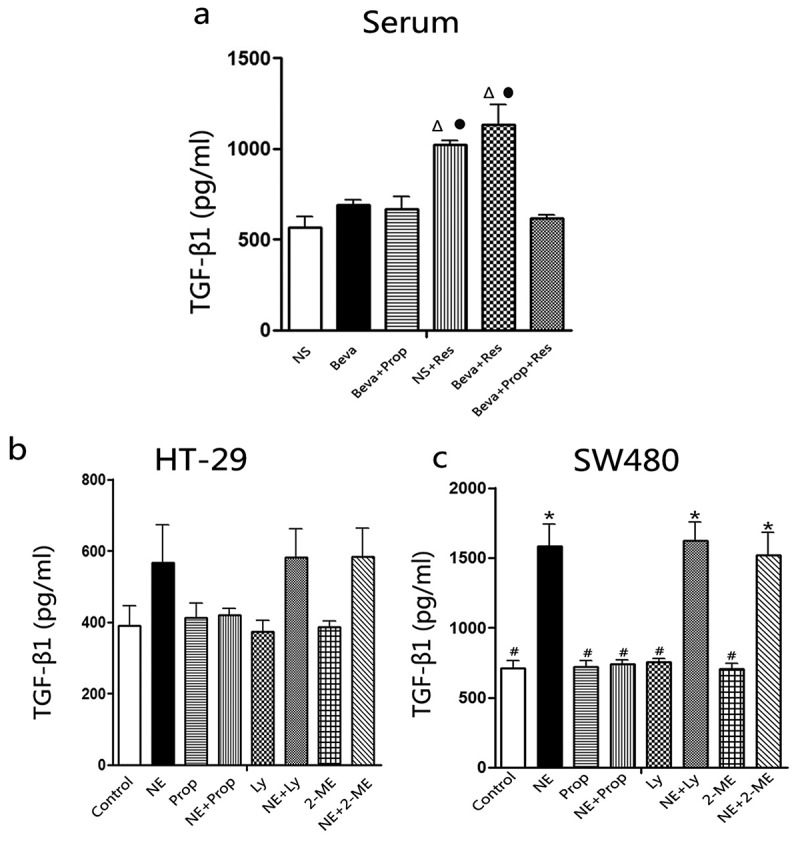
Chronic restraint stress and norepinephrine induced TGF-β1 secretion.

As described in the Introduction section, TGF-β1 can be induced by catecholamines. To verify whether CRS or NE could stimulate TGF-β1 secretion, TGF-β1 levels in mouse serum and culture supernatant were measured by ELISA (Figure 3a). CRS significantly increased TGF-β1 levels in mouse serum, regardless of whether the mice were treated with bevacizumab (Beva+Res group) or not (NS+Res group). However, in the Beva+Prop+Res group, the TGF-β1 level remained at the baseline level. There was no statistical significance among the NS, Beva, Beva+Prop, and Beva+Prop+Res groups. This indicated that CRS increased TGF-β1 *in vivo* in a β-AR-dependent manner.

CRS significantly increased TGF-β1 levels in mouse serum, and propranolol inhibited this elevation (a). Norepinephrine stimulated TGF-β1 secretion in SW480 cell culture supernatant and was blocked by propranolol (c). However, Ly2157299 or 2-ME did not reverse the NE effect. After NE treatment for 48 h, TGF-β1 levels were not significantly increased in HT-29 cells, but exhibited an increasing trend (b). ∆, *p* < .05 versus the NS group; •, *p* < .05 versus the Beva group; *, *p* < .05 versus the control group; #, *p* < .05 versus the NE group.

For the SW480 culture supernatant, TGF-β1 levels increased after NE-treatment for 48 h. Prop inhibited the NE effect, but Ly2157299 and 2-ME did not. TGF-β1 remained at the baseline level when the cells were only treated with Prop, Ly2157299, or 2-ME (Figure 3c). The TGF-β1 level in HT-29 cells did not significantly increase after NE-treatment for 48 h, but a trend was observed (Figure 3b). Interestingly, in another study, we found that TGF-β1 was significantly induced in HT-29 cells after NE-treatment for 72 h and Prop also played an inhibitive role.^30^

**Figure 4.**
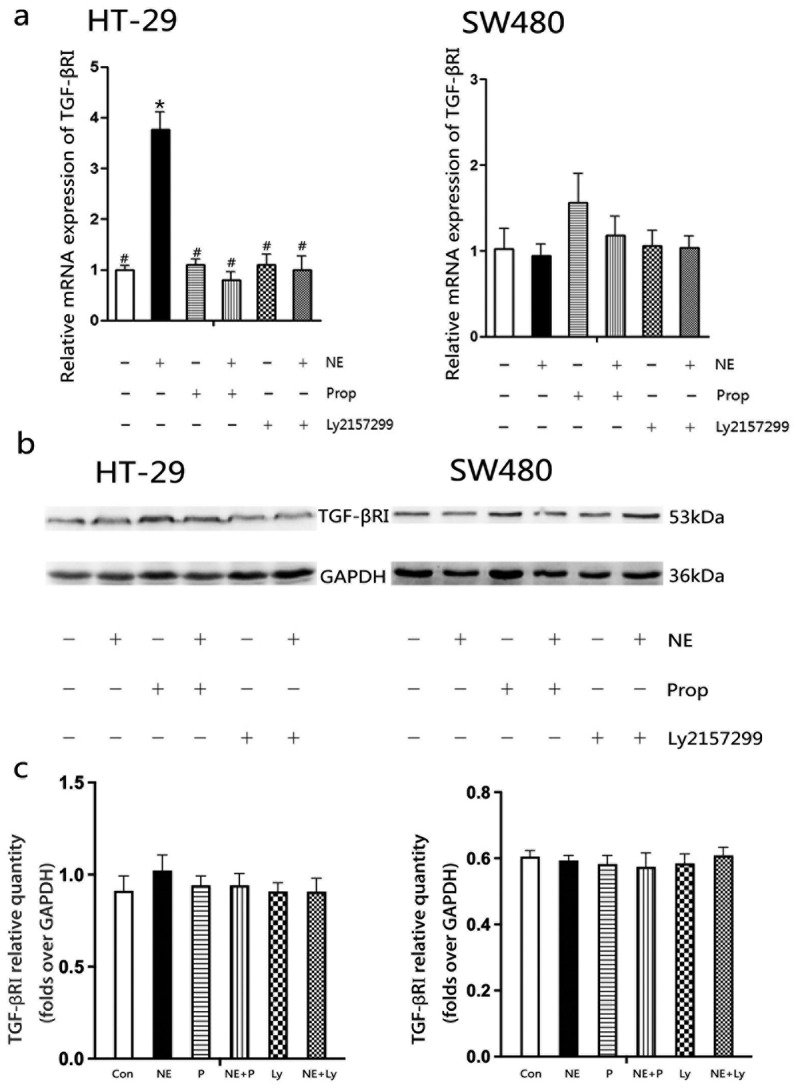
The mRNA and protein expression of TGF-βRI in HT-29 and SW480 cells after norepinephrine treatment.

The mRNA and protein expression of TGF-βRI were further analyzed because of its potential positive correlation with angiogenesis, in order to observe the effect of NE on TGF-βRI (Figure 4). After exposure to NE for 48 h, the mRNA expression of TGF-βRI was upregulated in HT-29 cells, while it remained at the baseline level in SW480 cells (Figure 4a). However, no significant difference was observed in HT-29 and SW480 cells at the protein level (Figure 4b). The quantification of proteins showed no statistically significant differences (Figure 4c).

NE induces TGF-βRI mRNA expression in HT-29 cells but not in SW480 cells (a). TGF-βRI protein expressions were similar in each group, both in HT-29 and SW480 cells (b). Quantification of proteins is presented in columns as the mean ± SD and showed no statistical difference (c). *p < .05, versus the control group; #*p* < .05, versus the NE group.

### 2.4. Norepinephrine promoted HIF-1α expression

**Figure 5.**
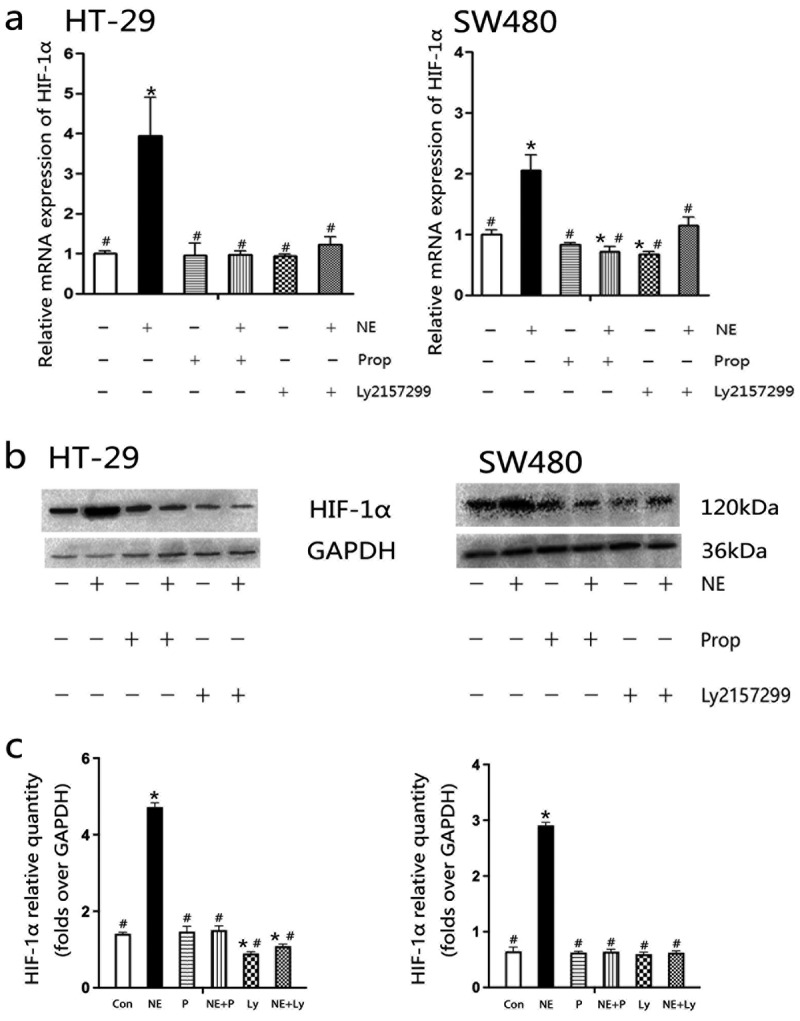
Propranolol or Ly2157299 blocked norepinephrine-induced mRNA and protein expression of HIF-1α.

HIF-1α is a downstream signal of TGF-β1 and plays a key role in VEGF secretion and tumor angiogenesis.^24,25,29^ Therefore, measurement of HIF-1α after NE treatment is critical. NE stimulated HIF-1α transcription in HT-29 and SW480 cells (Figure 5a). Both the β-AR antagonist Prop and TGF-βRI inhibitor Ly2157299 were able to block the effect of NE. Figure 5b shows that NE increased HIF-1α protein expression, and protein quantification (Figure 5c) showed similar results to mRNA. These results demonstrate that HIF-1α is a downstream signal of β-AR and TGF-β1. According to the results above, it also indicated that the β-AR/TGF-β1 signaling/HIF-1α might be a possible signaling pathway in this study.

After 48 h of treatment with NE, propranolol, or Ly2157299, mRNA (a) and protein (b) expression of HIF-1α were detected by RT-PCR and Western blotting, respectively. Quantification of HIF-1α protein is shown in the columns as the mean± SD (c). NE increased HIF-1α expression, whereas propranolol and Ly2157299 blocked this effect. *p < .05, versus the control group; #*p* < .05, versus the NE group.

### 2.5. Prop and Ly2157299 blocked norepinephrine-induced cancer cell migration and invasion

**Figure 6.**
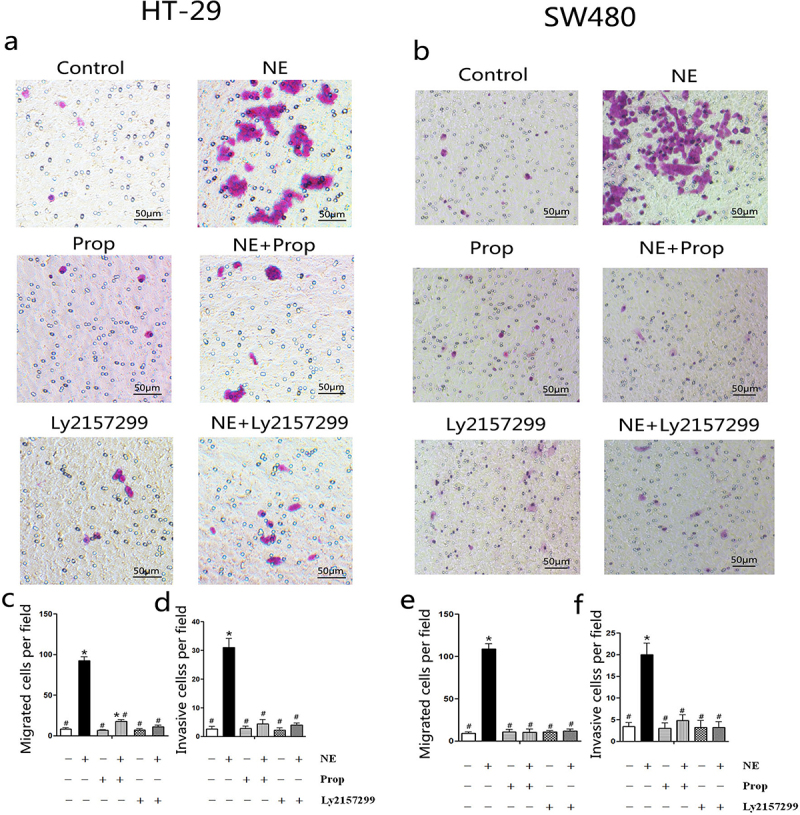
Norepinephrine stimulated HT-29 and SW480 cells migration and invasion.

NE and VEGF are associated with enhanced cancer cell migration and invasion.^15,19,31^ Transwell migration and Matrigel invasion assays were used to observe the migration and invasion abilities of the cells after NE treatment, respectively. As shown in the representative photographs (Figure 6a,b), the number of migratory (Figure 6c,e) and invasive cells (Figure 6d,f) in the NE-treated group was greater than that in the other groups. The results showed that NE stimulated the migration and invasion of HT-29 and SW480 cells. We also observed that Prop and Ly2157299 inhibited this process. This indicated that NE induced cell migration and invasion through β-AR and TGF-β signaling.

HT-29 and SW480 cells were treated with NE, propranolol, and Ly2157299. Representative images of cells that migrated to the lower membrane are shown in (HT-29) and (b) (SW480). The mean numbers of migratory (c and e) and invasive cells (d and f) were calculated from five random views and are presented in columns as mean± SD. Propranolol and Ly2157299 block NE-stimulated cell migration and invasion. *p < .05, versus the control group; #*p* < .05, versus the NE group.

## 3. Discussion

Chronic stress is a well-known risk factor for immune and cardiovascular diseases.^32,33^ It also increases the risk of tumorigenesis and progression via a variety of mechanisms, such as decreasing splenocytes, interferon-γ, tumor-infiltrating lymphocytes, and cytotoxic T-lymphocytes,^34^ attenuating P53 function^35^ and augmenting regulatory T cell (Treg) infiltration,^36^ leading to impaired anti-tumor immunity. On the other hand, promoting tumor angiogenesis is another mechanism by which chronic stress is involved.^14,37^ Retrospective clinical analyses have revealed that beta-blockers, including propranolol, decrease mortality and recurrence of breast cancer, ovarian cancer, and prostatic cancer and prolong disease-free survival and overall survival.^38–41^ We have reported that chronic stress-induced colorectal adenocarcinoma HT-29 cell EMT stimulated HT-29 cell migration and invasion, and weakened the anti-tumor effect of sunitinib *in vivo*.^15,30^ Unlike sunitinib, bevacizumab, a monoclonal antibody, was used in the present study. CRS exacerbated tumor load (Figure 1) and angiogenesis (Figure 2) in the HT-29 tumor model. Furthermore, propranolol blocks the effects of CRS. This indicated that CRS could impair the effect of bevacizumab via β-AR activation. Meanwhile, it might account for the unsatisfactory clinical results of single-agent bevacizumab in one aspect.

The CRS model is a classic and well-established framework for examining the effect of chronic stress on cancer in animal-based studies.^14,15,42^ It has been demonstrated to elicit depression-like or anxiety-like behaviors in mice,^43,44^ and to increase serum NE and VEGF in mice.^15,42^ Adenylyl cyclase is activated after NE binding to β-AR, leading to enhanced synthesis of cAMP. This activates the cAMP-dependent dependent protein kinase A (PKA), thereby initiating series of downstream molecules.^13,30,45^ NE is believed to promote TGF-β1 secretion.^20^ Our results also showed that CRS and NE elevated TGF-β1 levels in the serum and cell supernatant, respectively. This augmentation of TGF-β1 levels was inhibited by propranolol but not by Ly2157299 or 2-ME (Figure 3). TGF-β1 appears to be the downstream signaling pathway of β-AR, which can be induced through β-AR activation. Although there was no statistical difference in TGF-β1 levels between the NE-treated cells and the control at the time of 48 h in HT-29 cells, an increasing trend was discernible (Figure 3b). In another study, TGF-β1 levels were significantly increased by NE at 72 h^30^. In addition, at the 48-hour time point, TGF-βRI transcription was upregulated in HT-29 cells treated with NE, but no difference was observed at the protein level (Figure 4). Until the 96 h, TGF-βRI protein expression in HT-29 cells was significantly increased by NE.^30^ In contrast to the observations in HT-29 cells, no similar phenomenon was detected in the SW480 cells. We considered that cell specificity contributed to the different cellular responses to NE treatment.

TGF-β1 is believed to be associated with poor prognosis in colorectal cancer and is a predictor of hepatic metastasis.^23^ Evidences have shown that TGF-β1 weakens cancer immunotherapy,^46,47^ induces cancer cell EMT^30,48,49^ and promotes VEGF secretion.^50–52^ A clinical study reported that serum VEGF and TGF-β1 levels were positively correlated in cancer patients.^53^ As mentioned previously, HIF-1α is the downstream signaling pathway of TGF-β1 and plays a critical role in the stimulation of VEGF secretion.^24,25,29^ However, another study indicated that HIF-1α is an upstream signaling factor of TGF-β1.^54^ According to the results, propranolol inhibited NE-induced TGF-β1 secretion, but Ly2157299 and 2-ME did not (Figure 3c). Even in the HT-29 culture supernatant, an increase in TGF-β1 level was observed in the NE, NE+Prop, and NE + 2-ME groups (Figure 3b). It is possible that HIF-1α is a downstream signaling pathway of TGF-β1 in the present study. As shown in Figure 5, the mRNA and protein expression of HIF-1α was upregulated by NE treatment and was reversed by additional propranolol or Ly2157299 treatment. In another study, the β2-AR/HIF-1α/VEGF axis was found to regulate stress-induced tumor angiogenesis.^55^ Therefore, we hypothesized that β-AR/TGF-β signaling/HIF-1α/VEGF might be a signaling pathway involved in angiogenesis, which is stimulated by chronic stress. This may also influence the effect of bevacizumab.

EMT is a key factor in tumor invasion and metastasis.^56,57^ However, VEGF is an important determinant of the increased oncogenesis of cells undergoing EMT.^58,59^ Inhibition of the VEGF signaling pathway leads to the suppression of EMT. This is attributed to the role of VEGF in enhancing the migratory and invasive properties of MCF-7 cells.^60^ We found that NE could induce EMT in HT-29 cells and that β-AR/TGF-β1 signaling/p-Smad3/Snail and β-AR/TGF-β1 signaling/HIF-1α/Snail were two signaling pathways involved in this process.^30^ As shown in Figure 6, the migration and invasion of cells were enhanced after NE treatment. Therefore, we speculated that chronic stress could induce cancer cell EMT through several signaling pathways,^30,48,49^ including the promotion of angiogenesis and the further stimulation of EMT. However, the detailed mechanism requires further investigation.

## 4. Conclusions

Our study demonstrated that chronic stress stimulated tumor growth and angiogenesis and weakened the efficacy of bevacizumab, but was reversed by the β-AR antagonist propranolol. To our knowledge, this is the first study to investigate the effects of chronic stress on monoclonal antibodies. Norepinephrine, the most significant tumor-related SRHs, acts on β-AR and induces TGF-β1 and HIF-1α expression, leading to VEGF secretion. Taken together, β-AR/TGF-β1 signaling/HIF-1α/VEGF is a potential signaling pathway involved.

## 5. Material and methods

### 5.1. Reagents

Norepinephrine, propranolol, and 2-methoxyestradiol (2-ME, an HIF-1α inhibitor) were procured from Sigma-Aldrich (USA) and Enzo (Germany), respectively. Ly2157299, a TGF-β receptor Type I (TGF-βRI) kinase inhibitor, was supplied by Selleck (USA). Bevacizumab was obtained from Roche (Basel, Switzerland). Rabbit monoclonal antibodies against human HIF-1α and TGF-βRI were obtained from Cell Signaling (USA), and CD31 (rat anti-mouse monoclonal antibody) was purchased from BD Pharmingen (USA). Boster (China) provided the rabbit anti-human GAPDH (Glyceraldehyde-3-phosphate dehydrogenase), along with HRP-conjugated secondary rabbit anti-rat and goat anti-rabbit. TRIzol and the One Step SYBR® PrimeScript™ RT-PCR Kit were acquired from Thermo Fisher (USA) and TaKaRa (Japan), respectively. Human and mouse TGF-β1 ELISA kits (Neobioscience Technology Co., Ltd., China), Matrigel (BD Biosciences, USA), and 24-well Transwell (8-μm pore size, 6.5-mm diameter, Corning Costar Corporation, USA) were used.

### 5.2. Cell culture and treatment

The human colorectal adenocarcinoma cell lines HT-29 and SW480, kindly provided by the State Key Laboratory of Biotherapy (Sichuan University, Chengdu, China), were cultivated in RPMI1640 complete medium (Hyclone) supplemented with 10% fetal bovine serum (Hyclone), penicillin (100 U/mL), and streptomycin (10 mg/L) at 37°C in a humidified atmosphere of 5% CO_2_.

For the experiments *in vitro*, cells were allowed to reach 80% confluence, underwent serum starvation overnight, and were treated for 48 h according to the following six groups:1) serum-free culture medium (control); 2) 10 μM norepinephrine (NE); 3) 10 μM propranolol (Prop); 4) 10 μM NE +10 μM Prop (NE+P); 5) 10 μM Ly2157299 (Ly); 6) 10 μM NE +10 μM Ly2157299 (NE+Ly). All reagents were prepared in serum-free culture media to prevent interference from serum components. Propranolol and Ly2157299 were added to the culture media one hour prior to the addition of norepinephrine. Each group contained three replicates.

### 5.3. Establishment of tumor model and chronic restraint stress model

Five- to 7-week-old female athymic BALB/c nude mice were obtained from Beijing HFK Bioscience Co., Ltd. (Beijing, China) and were granted approval by the Sichuan University Animal Care and Use Committee. All mice were housed in a germ-free environment with food and water ad libitum, a regular 12-hour day/night cycle, and stable temperature ranging from 21–25°C. A tumorigenic challenge was initiated by inoculating the mice with 2.5 × 10^6^ HT-29 cells into the right flank. Upon the formation of palpable tumors, the mice were randomly distributed into six groups (five mice per group) and were administered the following treatments:1) NS, received normal saline 100 μL via intraperitoneal injection (i.p.) daily; 2) Beva, received normal saline 100 μL i.p. daily and bevacizumab i.p. twice a week; 3) Beva+Prop, received propranolol i.p. daily and bevacizumab i.p. twice a week; 4) NS+Res, received normal saline 100 μL i.p. daily and restraint stress daily; 5) Beva+Res, received normal saline 100 μL i.p. daily, restraint stress daily, and bevacizumab i.p. twice a week; and 6) Beva+Prop+Res, received propranolol i.p. daily, restraint stress daily, and bevacizumab i.p. twice a week. The dosages of bevacizumab and propranolol were 5 mg/kg/100 μL and 1 μmol/100 g/100 μL, respectively. The chronic restraint stress (CRS) model has been delineated in prior studies.^14,15,42^ Briefly, mice were constrained horizontally in well ventilated 50 mL conical centrifuge tubes (Corning, USA) for 2 h daily for 21 consecutive days. Mice in the tubes were able to breathe freely but were unable to move.

A caliper was utilized to monitor the longest dimension (length) and shortest dimension (width) of each tumor every 3 days. Tumor volume was estimated using the formula: volume (mm^3^) = length × width^2^ ×0.52. Following 21 consecutive days of restraint, all mice were anesthetized using chloral hydrate and subsequently sacrificed. Serum samples were collected and stored at −80°C for ELISA. Tumors in the right flank were carefully separated, weighed, immediately embedded in OCT compound (Sakura Finetek USA, USA), and conserved at −80°C for immunohistochemistry.

### 5.4. Immunohistochemistry for CD31

To evaluate vessel densities in tumor tissues, frozen tumors were sliced into 5 μm at −20°C and then stained with monoclonal rat anti-mouse CD31, a marker for murine endothelial cell. Briefly, frozen sections were fixed in cold acetone for 20 min, followed by three washed in phosphate-buffered saline (PBS). After incubation with 3% H_2_O_2_ for 10 min and washing with PBS, sections were incubated with CD31 (1:400) at 4°C overnight. The next day, the sections underwent a final PBS wash, followed by a 2-hour incubation with a rabbit anti-rat antibody at a temperature of 37°C. The sections were examined using a microscope. The MVD of each sample was calculated by determining the average count of immunoreactive endothelial cells in five random fields (200×).

### 5.5. Assessment of TGF-β1

HT-29 and SW480 cells were treated for 48 h according to the following eight groups:1) control, 2) 10 μM NE; 3) 10 μM Prop; 4) 10 μM NE +10 μM Prop, 5) 10 μM Ly; 6) 10 μM NE +10 μM Ly, 7) 10 μM 2-ME; 8) 10 μM NE +10 μM 2-ME. Following the treatment, the supernatant from the cell cultures was harvested for ELISA analysis. The mouse serum described above was stored at −80°C for ELISA. Adhering to the guidelines provided by the manufacturer, the levels of TGF-β1 in the mouse serum and culture supernatant were detected using mouse and human TGF-β1 ELISA kits, respectively. The luminescence plate reader (PerkinElmer, USA) was used to read the plates with a wavelength setting of 450 nm. The concentration of each sample was calculated by using a standard curve. Each sample was tested in triplicates.

### 5.6. Cell migration and invasion assay

To explore the impact of NE on the migration and invasive capabilities of cancer cells, the Transwell migration assay and the Matrigel invasion assay were performed as described in previous studies.^15^ HT-29 and SW480 cells, which had been deprived of serum, were suspended in serum-free medium at a density of 2.5 × 10^6^/mL. To serve as a chemoattractant, 500 μL of medium containing 10% fetal bovine serum was added to each well of lower chamber. A volume of 100 μL cell suspension (HT-29 or SW480, total number of each well was 2.5 × 10^5^) was transferred to the upper wells. Subsequently, the cells were subjected to treatment with NE, Prop, or Ly2157299, in accordance with the six groups outlined for cell culture and treatment protocols, and were subsequently incubated for 12 hours at 37°C

Cotton swabs were used to remove cells from the upper surface of the separating membranes. After immersion in 4% paraformaldehyde for 30 min, the separated membranes were stained with 0.1% crystal violet (Beyotime, China) for 20 min. The migratory cells remained on the lower surface of the separating membranes and were stained purple. Cell migration was evaluated by averaging the total number of migratory cells from five randomly selected views under a microscope with a 200× magnification.

The Matrigel invasion assay followed a similar procedure to the Transwell migration assay. The upper chambers of the transwell were coated with matrigel (100 μL of 1.0 mg/mL) beforehand. Medium (500 μL) supplemented with 10% fetal bovine serum was added to each lower chamber. Serum-starved 5 × 10^5^/100 μL HT-29 or SW480 cells were seeded in the upper chambers, exposed to treatments with NE, Prop, or Ly2157299 according to the groups, and incubated for 24 h at 37°C. The remaining procedure was the same as that used in the Transwell migration assay.

### 5.7. Real-time quantitative PCR (RT-PCR)

Following the RNA extraction protocols, total RNA from HT-29 and SW480 cells in the six groups described in Cell culture and treatment was isolated using TRIzol reagent (15596018, Thermo Fisher). RT-PCR was conducted using the One Step SYBR® PrimeScript™ RT-PCR Kit and the CFX 96™ Real-Time System in a C1000™ Thermal Cycler (Bio-Rad, USA). The sequences of the primers used in this study were as follows:

TGF-βRI sense 5′-GCTGTGAAGCCTTGAGAGTAATGG-3′,
anti-sense 5′-TTCCTGTTGACTGAGTTGCGATAA-3′;
**Accession**:NM_001130916.
  HIF-1α sense 5′-ACTGCACAGGCCACATTCACG-3′,
anti-sense 5′-GGTTCACAAATCAGCACCAAGC-3′;
**Accession**:NM_001243084.
GAPDH sense 5-CTTTGGTATCGTGGAAGGACTC-3′,
anti-sense 5′-GTAGAGGCAGGGATGATGTTCT-3′.
**Accession**:NM_001256799.

GAPDH was used as an internal positive control. RT-PCR was executed according to the manufacturer’s guidelines. Each sample was processed at least in triplicate, and the relative mRNA expression levels of TGF-β1 and HIF-1α were calculated using the following formula: relative expression = 2^−(ΔCt)^, ΔCt= (Ct gene of interest – Ct GAPDH).

### 5.8. Western blot analysis

Proteins of the cells in the six groups described in the “Cell culture and treatment” section were extracted for western blot analysis. Briefly, cells were washed and collected by gentle scraping with cold PBS. The suspension was centrifuged and the precipitate was resuspended in RIPA buffer (150 mM NaCl, 0.1% SDS, 1 mM sodium orthovanadate, 1% TritonX-100, 0.5% sodium deoxycholate, 50 mM Tris-base, 10 mM sodium fluoride, 1% phosphatase inhibitor cocktail, and 1% protease inhibitor cocktail). The mixture was incubated on ice for 30 min, followed by a centrifugation step to remove the debris. Approximate 10–30 μg of proteins were resuspended in SDS-PAGE loading buffer, separated on polyacrylamide gel and electro-transferred into nitrocellulose membranes (Millipore, USA). The membranes were blocked with 5% nonfat milk for 1 h at room temperature and incubated with rabbit monoclonal antibodies against human HIF-1α (1:1000) or TGF-βRI (1:1000) at 4°C overnight. After extensive washing with TBST (Tris Buffer saline containing 0.1% Tween-20 (TBST) to remove unbound primary antibodies, the membranes were incubated with HRP-conjugated secondary goat anti-rabbit antibody, diluted to 1:3000, for a period of 2 hours at room temperature. The membranes were washed again with TBST, and the immunoreactive bands were visualized using Chemiluminescent HRP Substrate (Millipore, USA) and analyzed using Image-Pro Plus 6.0.

### 5.9. Statistical analysis

The data are presented as mean ± standard deviation (SD). All statistical analyses were performed using SPSS version 23.0. One-way analysis of variance (ANOVA) and repeated-measures ANOVA were used to analyze the differences between groups. A two-sided *p* < .05 was considered statistically significant.

## Data Availability

The data supporting the findings of this study are available from the corresponding author, JY, upon reasonable request. The primer sequences used in this study were obtained from the National Center for Biotechnology Information (NCBI): https://www.ncbi.nlm.nih.gov/guide/dna-rna/.
